# The mediating role of positive mental attitude in the relationship between mental toughness and athlete burnout: a cross-sectional study

**DOI:** 10.3389/fpubh.2025.1741676

**Published:** 2026-01-16

**Authors:** Łukasz Bojkowski

**Affiliations:** Department of Psychology, Poznan University of Physical Education, Poznań, Poland

**Keywords:** mental toughness, health behaviors, athlete burnout, mental health prevention in sport, mediation analysis

## Abstract

**Introduction:**

Athlete burnout poses a significant threat to both wellbeing and performance. Mental toughness is recognized as a key protective factor. However, the mechanisms underlying its relationship with burnout remain insufficiently understood. The present study aimed to examine whether positive mental attitude—a component of health-related behaviours—mediates the relationship between mental toughness and athlete burnout.

**Methods:**

A cross-sectional study was conducted among 456 athletes aged 18–30 years. The following Polish-adapted instruments were employed: the Sports Mental Toughness Questionnaire (SMTQ-P), the Health Behavior Inventory (IZZ)—Positive Mental Attitude subscale, and the Athlete Burnout Questionnaire (ABQ). Mediation analyses were performed using bootstrapping (5000 samples) to test the indirect effects of mental toughness (total score and subscales) on burnout (total and subscale scores) via positive mental attitude.

**Results:**

Positive mental attitude significantly mediated the relationship between overall mental toughness and total burnout. At the level of mental toughness subscales, mediation effects were observed for self-confidence and effectiveness in predicting emotional and physical exhaustion and reduced sense of accomplishment. In contrast, emotional control and task execution showed no significant indirect effects through positive mental attitude. No mediation was found for sport devaluation.

**Conclusion:**

Positive mental attitude partially explains the relationship between mental toughness and lower levels of burnout—particularly in the domains of emotional and physical exhaustion and reduced sense of accomplishment. Preventive and educational programs should focus on simultaneously enhancing key components of mental toughness (e.g., self-confidence, effectiveness) and fostering a positive mental attitude to support athletes’ psychological resilience and long-term engagement in sport.

## 1 Introduction

The issue of burnout in sport is a multidimensional phenomenon. It concerns individuals engaged in sporting activities—athletes, coaches (1), and those working in sport-related professions (2, 3). Conceptually, athlete burnout is defined as a state characterized by three key dimensions (4, 5):

1. Emotional and physical exhaustion—a sense of fatigue resulting from the psychological and physical demands associated with training and competition.
2. Reduced sense of accomplishment—feelings of ineffectiveness and a tendency to evaluate one’s performance and achievements negatively.
3. Sport devaluation—a detached or negative attitude toward one’s sport, accompanied by a loss of interest and diminished quality of engagement.

The phenomenon of burnout is influenced by several contributing factors, including chronic stress experienced by athletes—which may ultimately lead to sport withdrawal (6, 7)—excessive performance demands (8, 9), insufficient recovery (1), and interpersonal conflicts (10). In turn, the negative health consequences of this syndrome—often discussed in relation to one’s professional activity—commonly include type 2 diabetes, cardiovascular diseases, depressive symptoms, reduced job satisfaction (9), insomnia (11), and attention disorders (12).

As outlined above, athlete burnout is a widespread issue that can adversely affect not only the athletes themselves but also the broader sporting environment. It impacts both psychological and physical wellbeing, as well as overall health. Consequently, increasing attention is being paid to education aimed at understanding the processes leading to burnout at the individual level (with consideration of personal resources) (13), effective stress management, and the development of organizational resources related to specific working conditions (14). More specifically, strategies proposed to enhance the wellbeing of individuals at risk of—or currently experiencing—burnout include fostering a greater sense of personal accomplishment and reducing depersonalization within the sporting context (15), limiting avoidant coping strategies (16), managing psychological and situational factors such as amotivation, fatigue, and dissatisfaction with performance outcomes, as well as promoting autonomy and supportive social relationships (17–19).

In the context of athletic performance—including the prevention and management of burnout—strategies aimed at developing mental toughness may also prove beneficial. Mental toughness is defined as the ability to consistently achieve high performance despite challenges, stressors, external pressure, or significant adversity (20), or as the capacity to maintain optimal functioning in the face of difficulties (21, 22). For instance, a study by Lu et al. (23) found that higher levels of mental toughness among athletes, combined with perceived support from coaches, may protect against burnout arising from elevated stress levels. Similar findings were reported by Madigan and Nicholls (24) and Gerber et al. (25). These observations are particularly important given the growing recognition that mental toughness can also be viewed as a trainable skill shaped by environmental factors (21) and, therefore, consciously and deliberately developed (22).

At the same time, a relationship has been observed between the development of mental toughness and positive mental attitude, understood as a dimension of health-related behavior (26). Considering the detrimental effects of burnout, this approach aims not only to examine the relationships between the selected variables but also to emphasize the health-promoting role and practical relevance of developing mental toughness in sport. Previous research indicates that athletes with higher levels of mental toughness tend to exhibit a more positive mental attitude (27, 28), which contributes to their psychological wellbeing (29) and satisfaction with interpersonal relationships in their immediate environment (30). Importantly, relationships with coaches can significantly reduce the risk of burnout, as demonstrated in a sample of Norwegian athletes (31).

Accordingly, this study aimed to examine how different dimensions of mental toughness—specifically self-confidence, effectiveness, emotional control, and task execution (32)—are related to athlete burnout, taking into account the mediating role of positive mental attitude (26). Burnout was assessed using the Polish adaptation (33) of the Athlete Burnout Questionnaire (ABQ), originally developed by Raedeke and Smith.

## 2 Materials and methods

### 2.1 Participants

A total of 456 Polish athletes aged 18–30 years (*M* = 20.91, SD = 2.60) participated in the study, including 213 women (45.7%) and 243 men (53.3%). The participants’ training experience ranged from 12 to 276 months (*M* = 105.95, SD = 54.91). The sample included student-athletes from the Poznan University of Physical Education, as well as competitors representing various sports disciplines such as: short- and long-distance running, Brazilian jiu-jitsu, fitness, equestrianism, field hockey, kayaking, cycling, basketball, bodybuilding, football, handball, swimming, volleyball, dance, triathlon, powerlifting, rowing, sport climbing, and wrestling. The inclusion criteria were as follows: legal adulthood (≥18 years), age not exceeding 30 years, participation in at least three training sessions per week, and active representation of a sports club or organization in competitions of at least national level within the past 12 months.

The study was conducted between October 2023 and October 2024. The research procedure was reviewed by the Bioethics Committee of the Poznan University of Medical Sciences in Poznań (Poland), which stated on 5 October 2023 confirming that the study did not constitute a medical experiment and therefore did not require ethical approval under current regulations. The researchers complied with all recommendations of the Committee. The study was anonymous and conducted in accordance with the Declaration of Helsinki, under the direct supervision of a psychologist.

### 2.2 Measurement tools

1 Sports Mental Toughness Questionnaire—Polish version (SMTQ-P)—a psychometric instrument designed to measure mental toughness within the sports context, originally developed by Sheard and colleagues. The Polish adaptation was prepared by Guszkowska et al. (32), taking into account the specific characteristics of the Polish athletic population.
  The Polish version (which differs notably from the original tool developed by Sheard, Golby, and van Wersch) consists of 13 items rated on a 4-point Likert scale ranging from 1 (“strongly disagree”) to 4 (“strongly agree”). The questionnaire measures an overall mental toughness score and four key components relevant to athletes’ functioning in sport: self-confidence (largely corresponding to the Confidence subscale from the original version), effectiveness, emotional control, and task execution (the latter subscales include items either derived from or combined across two of the original scales) (32).
  The SMTQ-P demonstrates satisfactory psychometric properties, with Cronbach’s alpha coefficients ranging from 0.64 to 0.79, indicating good overall reliability and acceptable internal consistency of the subscales—particularly suitable for screening and diagnostic use in applied sport settings. Theoretical validity of the instrument has been confirmed through correlational analyses (32).

2 Health Behavior Inventory (IZZ)—a psychometric instrument developed by Juczyński (26) to assess habits and behaviors related to the protection and enhancement of health.
  The IZZ consists of 25 statements referring to various aspects of everyday functioning that promote health. Respondents indicate how often each behavior occurs in their daily life using a 5-point Likert scale (from 1—“almost never” to 5—“almost always”). The inventory allows for the calculation of an overall health behavior index and four subscales, including “positive mental attitude”—which encompasses coping strategies, optimism, the ability to control negative emotions, and habits related to maintaining psychological wellbeing. This subscale was used in this study. A high score reflects a conscious effort to maintain mental health, which is associated with lower risk of psychosomatic symptoms and greater resilience to stress (26).
  The IZZ demonstrates good reliability, with a Cronbach’s alpha of 0.85 for the overall index and 0.60–0.65 for the “positive mental attitude” subscale. Theoretical validity has been confirmed through correlational studies with other measures of health-related constructs (26).

3 Athlete Burnout Questionnaire (ABQ)—an instrument designed to assess burnout among athletes of various ages, including both men and women participating in individual and team sports. The questionnaire was originally developed by Raedeke and Smith, and its Polish adaptation was prepared by Cichosz-Dziadura et al. (33).
  The Polish version of the ABQ consists of 15 items rated on a 5-point Likert scale ranging from 1 (“I almost never feel this way”) to 5 (“I feel this way most of the time”). The questionnaire measures an overall burnout score as well as three core dimensions, which can also be analyzed separately: emotional and physical exhaustion, reduced sense of athletic accomplishment, and sport devaluation (33).
  The Polish adaptation of the ABQ demonstrates very good psychometric properties, with Cronbach’s alpha coefficients of 0.81 for emotional and physical exhaustion, 0.69 for reduced sense of accomplishment, and 0.67 for sport devaluation—indicating good overall reliability and acceptable internal consistency across subscales. Theoretical validity was confirmed through significant correlations with related constructs, such as the Sport Motivation Scale (SMS-6) (33).

### 2.3 Statistical analysis

To determine the mediating role of positive mental attitude in the relationship between mental toughness and athlete burnout, mediation analyses were conducted. Mental toughness and its subscales were treated as independent variables, while burnout and its components served as dependent variables, and positive mental attitude as the mediator. A mediation effect was considered statistically significant when the indirect effect—representing the relationship between the independent variable, the mediator, and the dependent variable—was statistically significant. Statistical significance was evaluated using the bootstrap percentile method, and effects were considered significant when the 95% confidence interval did not include zero. Confidence intervals were estimated using 5000 bootstrap samples (34). All computations were performed using Jamovi software (version 2.6.26).

## 3 Results

First, a mediation analysis was conducted with the overall mental toughness score as the independent variable and overall athlete burnout as the dependent variable. Subsequently, separate analyses were performed for the individual subscales of both constructs.

Positive mental attitude significantly mediated the relationship between mental toughness and athlete burnout (*b* = −0.0190, 95% confidence interval [CI −0.0303; −0.0087], *β* = −0.0689). Mental toughness was positively associated with positive mental attitude (*b* = 0.0352, 95% CI [0.0259; 0.0446], *β* = 0.3517), which in turn was negatively associated with athlete burnout (*b* = −0.5384, 95% CI [−0.8129; −0.2639], *β* = −0.1959) (Table 1).

**Table 1:** Positive mental attitude as a mediator in the relationship between mental toughness and athlete burnout—results of the mediation analysis.

Mediators models *N* = 456
Mental Toughness ⟹ Positive Mental Attitude ⟹ Athlete Burnout

				95% C.I.		*β*	*z*	*p*
Type	Effect	Estimate	SE	Lower	Upper			
Indirect	MT ⟹ PMA ⟹ AB	−0.0190	0.0049	−0.0303	−0.0087	−0.0689	−3.85	<0.001
Component	MT ⟹ PMA	0.0352	0.0044	0.0259	0.0446	0.3517	8.02	<0.001
	PMA ⟹ AB	−0.5384	0.1228	−0.8129	−0.2639	−0.1959	−4.38	<0.001
Direct	MT ⟹ AB	−0.0939	0.0123	−0.1255	−0.0625	−0.3412	−7.63	<0.001
Total	MT ⟹ AB	−0.1129	0.0118	−0.1422	−0.0825	−0.4101	−9.59	<0.001

Confidence intervals computed with method: bootstrap percentiles 5000 (interval 95%). Betas are completely standardized effect size. MT, Mental Toughness; PMA, Positive Mental Attitude; AB, Athlete Burnout.

**Figure 1:**
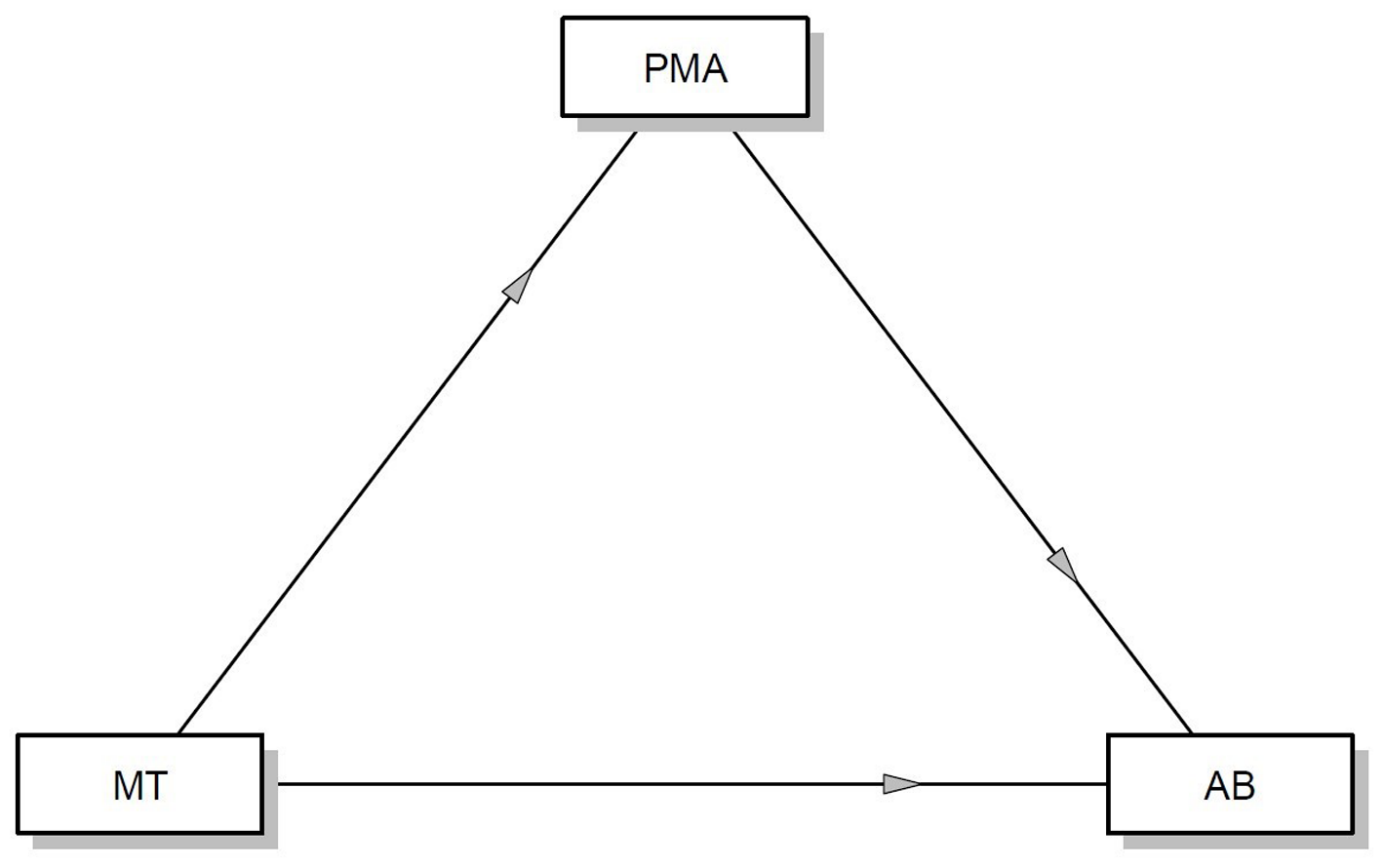
Positive mental attitude as a mediator between mental toughness and athlete burnout.

Below is a graphical representation of the mediation model, where mental toughness serves as the independent variable, positive mental attitude as the mediator, and athlete burnout as the dependent variable (Figure 1).

Positive mental attitude was found to be a significant mediator in the relationship between self-confidence (*b* = −0.0056, 95% CI [−0.0116; −0.0001], *β* = −0.0239) and effectiveness (*b* = −0.0105, 95% CI [−0.0214; −0.0028], *β* = −0.0392) with emotional and physical exhaustion. Self-confidence (*b* = 0.0266, 95% CI [0.0042; 0.0490], *β* = 0.1277) and effectiveness (*b* = 0.0501, 95% CI [0.0179; 0.0821], *β* = 0.2090) were positively associated with positive mental attitude, which in turn was negatively associated with emotional and physical exhaustion (*b* = −0.2098, 95% CI [−0.3221; −0.0973], *β* = −0.1874). However, positive mental attitude did not mediate the relationship between emotional control (*b* = −0.0095, 95% CI [−0.0232; 0.0001], *β* = −0.0204) or task execution (*b* = −0.0001, 95% CI [−0.0111; 0.0111], *β* = −0.0001) and emotional and physical exhaustion among athletes (Table 2).

**Table 2:** Positive mental attitude as a mediator in the relationship between components of mental toughness and emotional and physical exhaustion (athlete burnout subscale)—results of the mediation analysis.

Mediators models *N* = 456
Self-Confidence y ⟹ Positive Mental Attitude ⟹ Emotional and Physical Exhaustion Effectiveness ⟹ Positive Mental Attitude ⟹ Emotional and Physical Exhaustion Emotional Control ⟹ Positive Mental Attitude ⟹ Emotional and Physical Exhaustion Task Execution ⟹ Positive Mental Attitude ⟹ Emotional and Physical Exhaustion

				95% C.I.		*β*	*z*	*p*
Type	Effect	Estimate	SE	Lower	Upper			
Indirect	SC ⟹ PMA ⟹ EX	−0.0056	0.0026	−0.0116	−0.0001	−0.0239	−2.1714	0.030
	EF ⟹ PMA ⟹ EX	−0.0105	0.0039	−0.0214	−0.0028	−0.0392	−2.6879	0.007
	EC ⟹ PMA ⟹ EX	−0.0095	0.0052	−0.0232	0.0001	−0.0204	−1.8239	0.068
	TE ⟹ PMA ⟹ EX	−0.0001	0.0052	−0.0111	0.0111	−0.0001	−0.0746	0.941
Component	SC ⟹ PMA	0.0266	0.0104	0.0042	0.0490	0.1277	2.5583	0.011
	EF ⟹ PMA	0.0501	0.0141	0.0179	0.0821	0.2090	3.5553	<0.001
	EC ⟹ PMA	0.0455	0.0224	−0.0037	0.0945	0.1090	2.0357	0.042
	TE ⟹ PMA	0.0018	0.0246	−0.0474	0.0505	0.0036	0.0746	0.941
	PMA ⟹ EX	−0.2098	0.0511	−0.3221	−0.0973	−0.1874	−4.1066	<0.001
Direct	SC ⟹ EX	−0.0037	0.0114	−0.0283	0.0211	−0.0160	−0.3274	0.743
	EF ⟹ EX	−0.0186	0.0156	−0.0541	0.0161	−0.0694	−1.1941	0.232
	EC ⟹ EX	−0.1054	0.0245	−0.1589	−0.0526	−0.2256	−4.3048	<0.001
	TE ⟹ EX	−0.0612	0.0268	−0.1188	−0.0037	−0.1068	−2.2811	0.023
Total	SC ⟹ EX	−0.0093	0.0116	−0.0339	0.0149	−0.0400	−0.8061	0.420
	EF ⟹ EX	−0.0291	0.0157	−0.0661	0.0064	−0.1085	−1.8581	0.063
	EC ⟹ EX	−0.1150	0.0249	−0.1696	−0.0634	−0.2460	−4.6259	<0.001
	TE ⟹ EX	−0.0616	0.0273	−0.1193	−0.0040	−0.1074	−2.2516	0.024

Confidence intervals computed with method: bootstrap percentiles 5000 (interval 95%). Betas are completely standardized effect size. SC, Self-Confidence; EF, Effectiveness; EC, Emotional Control; TE, Task Execution; PMA, Positive Mental Attitude; EX, Emotional and Physical Exhaustion.

Positive mental attitude was found to be a significant mediator in the relationship between self-confidence (*b* = −0.0058, 95% CI [−0.0117; −0.0001], *β* = −0.0271) and effectiveness (*b* = −0.0109, 95% CI [−0.0215; −0.0031], *β* = −0.0443) with reduced sense of athletic accomplishment. Self-confidence (*b* = 0.0266, 95% CI [0.0050; 0.0484], *β* = 0.1277) and effectiveness (*b* = 0.0501, 95% CI [0.0179; 0.0829], *β* = 0.2090) were positively associated with positive mental attitude, which was negatively related to reduced sense of athletic accomplishment (*b* = −0.2179, 95% CI [−0.3129; −0.1195], *β* = −0.2119). At the same time, positive mental attitude did not mediate the relationship between emotional control (*b* = −0.0099, 95% CI [−0.0222; 0.0001], *β* = −0.0231) or task execution (*b* = −0.0001, 95% CI [−0.0113; 0.0111], *β* = −0.0001) and reduced sense of athletic accomplishment among athletes (Table 3).

**Table 3:** Positive mental attitude as a mediator in the relationship between components of mental toughness and reduced sense of athletic accomplishment (athlete burnout subscale)—results of the mediation analysis.

Mediators models *N* = 456
Self-Confidence ⟹ Positive Mental Attitude ⟹ Reduced Sense of Athletic Accomplishment Effectiveness ⟹ Positive Mental Attitude ⟹ Reduced Sense of Athletic Accomplishment Emotional Control ⟹ Positive Mental Attitude ⟹ Reduced Sense of Athletic Accomplishment Task Execution ⟹ Positive Mental Attitude ⟹ Reduced Sense of Athletic Accomplishment

				95% C.I.		*β*	*z*	*p*
Type	Effect	Estimate	SE	Lower	Upper			
Indirect	SC ⟹ PMA ⟹ RSA	−0.0058	0.0026	−0.0117	−0.0001	−0.0271	−2.2568	0.024
	EF ⟹ PMA ⟹ RSA	−0.0109	0.0038	−0.0215	−0.0031	−0.0443	−2.8552	0.004
	EC ⟹ PMA ⟹ RSA	−0.0099	0.0053	−0.0222	0.0001	−0.0231	−1.8736	0.061
	TE ⟹ PMA ⟹ RSA	−0.0001	0.0054	−0.0113	0.0111	−0.0001	−0.0746	0.941
Component	SC ⟹ PMA	0.0266	0.0104	0.0050	0.0484	0.1277	2.5583	0.011
	EF ⟹ PMA	0.0501	0.0141	0.0179	0.0829	0.2090	3.5553	<0.001
	EC ⟹ PMA	0.0455	0.0224	−0.0038	0.0959	0.1090	2.0357	0.042
	TE ⟹ PMA	0.0018	0.0246	−0.0475	0.0519	0.0036	0.0746	0.941
	PMA ⟹ RSA	−0.2179	0.0455	−0.3129	−0.1195	−0.2119	−4.7914	<0.001
Direct	SC ⟹ RSA	−0.0332	0.0102	−0.0554	−0.0115	−0.1545	−3.2556	0.001
	EF ⟹ RSA	−0.0408	0.0139	−0.0719	−0.0080	−0.1654	−2.9388	0.003
	EC ⟹ RSA	−0.0250	0.0218	−0.0699	0.0194	−0.0583	−1.1477	0.251
	TE ⟹ RSA	−0.0527	0.0239	−0.1096	0.0048	−0.1002	−2.2080	0.027
Total	SC ⟹ RSA	−0.0390	0.0104	−0.0606	−0.0174	−0.1816	−3.7554	<0.001
	EF ⟹ RSA	−0.0517	0.0140	−0.0832	−0.0182	−0.2097	−3.6813	<0.001
	EC ⟹ RSA	−0.0349	0.0223	−0.0814	0.0118	−0.0814	−1.5689	0.117
	TE ⟹ RSA	−0.0531	0.0245	−0.1088	0.0046	−0.1009	−2.1684	0.030

Confidence intervals computed with method: bootstrap percentiles 5000 (interval 95%). Betas are completely standardized effect size. SC, Self-Confidence; EF, Effectiveness; EC, Emotional Control; TE, Task Execution; PMA, Positive Mental Attitude; RSA, Reduced Sense of Athletic Accomplishment.

Positive mental attitude did not significantly mediate any of the relationships between the components of mental toughness (self-confidence, effectiveness, emotional control, task execution) and sport devaluation (a subscale of athlete burnout). For none of the tested associations between variables did the interaction effect reach statistical significance, as all confidence intervals included zero. This indicates that positive mental attitude did not serve as a mediator in the relationships examined (Table 4).

**Table 4:** Positive mental attitude as a mediator in the relationship between components of mental toughness and sport devaluation (athlete burnout subscale)—results of the mediation analysis.

Mediators models *N* = 456
Self-Confidence ⟹ Positive Mental Attitude ⟹ Sport Devaluation Effectiveness ⟹ Positive Mental Attitude ⟹ Sport Devaluation Emotional Control ⟹ Positive Mental Attitude ⟹ Sport Devaluation Task Execution ⟹ Positive Mental Attitude ⟹ Sport Devaluation

				95% C.I.		*β*	*z*	*p*
Type	Effect	Estimate	SE	Lower	Upper			
Indirect	SC ⟹ PMA ⟹ SD	−0.0029	0.0019	−0.0078	0.0001	−0.0123	−1.5743	0.115
	EF ⟹ PMA ⟹ SD	−0.0055	0.0032	−0.0139	0.0001	−0.0201	−1.7413	0.082
	EC ⟹ PMA ⟹ SD	−0.0050	0.0035	−0.0149	0.0013	−0.0105	−1.4257	0.154
	TE ⟹ PMA ⟹ SD	−0.0001	0.0027	−0.0067	0.0064	−0.0001	−0.0745	0.941
Component	SC ⟹ PMA	0.0266	0.0104	0.0045	0.0489	0.1277	2.5583	0.011
	EF ⟹ PMA	0.0501	0.0141	0.0172	0.0829	0.2090	3.5553	<0.001
	EC ⟹ PMA	0.0455	0.0224	−0.0057	0.0953	0.1090	2.0357	0.042
	TE ⟹ PMA	0.0018	0.0246	−0.0471	0.0515	0.0036	0.0746	0.941
	PMA ⟹ SD	−0.1105	0.0553	−0.2317	0.0128	−0.0960	−1.9973	0.046
Direct	SC ⟹ SD	−0.0152	0.0124	−0.0414	0.0124	−0.0631	−1.2239	0.221
	EF ⟹ SD	−0.0098	0.0169	−0.0477	0.0270	−0.0356	−0.5811	0.561
	EC ⟹ SD	−0.0556	0.0265	−0.1047	−0.0057	−0.1156	−2.0939	0.036
	TE ⟹ SD	−0.0785	0.0291	−0.1368	−0.0190	−0.1332	−2.7006	0.007
Total	SC ⟹ SD	−0.0181	0.0124	−0.0429	0.0076	−0.0754	−1.4639	0.143
	EF ⟹ SD	−0.0153	0.0167	−0.0519	0.0203	−0.0556	−0.9166	0.359
	EC ⟹ SD	−0.0606	0.0266	−0.1115	−0.0089	−0.1260	−2.2813	0.023
	TE ⟹ SD	−0.0787	0.0292	−0.1407	−0.0196	−0.1335	−2.6929	0.007

Confidence intervals computed with method: bootstrap percentiles 5000 (interval 95%). Betas are completely standardized effect size. SC, Self-Confidence; EF, Effectiveness; EC, Emotional Control; TE, Task Execution; PMA, Positive Mental Attitude; SD, Sport Devaluation.

**Figure 2:**
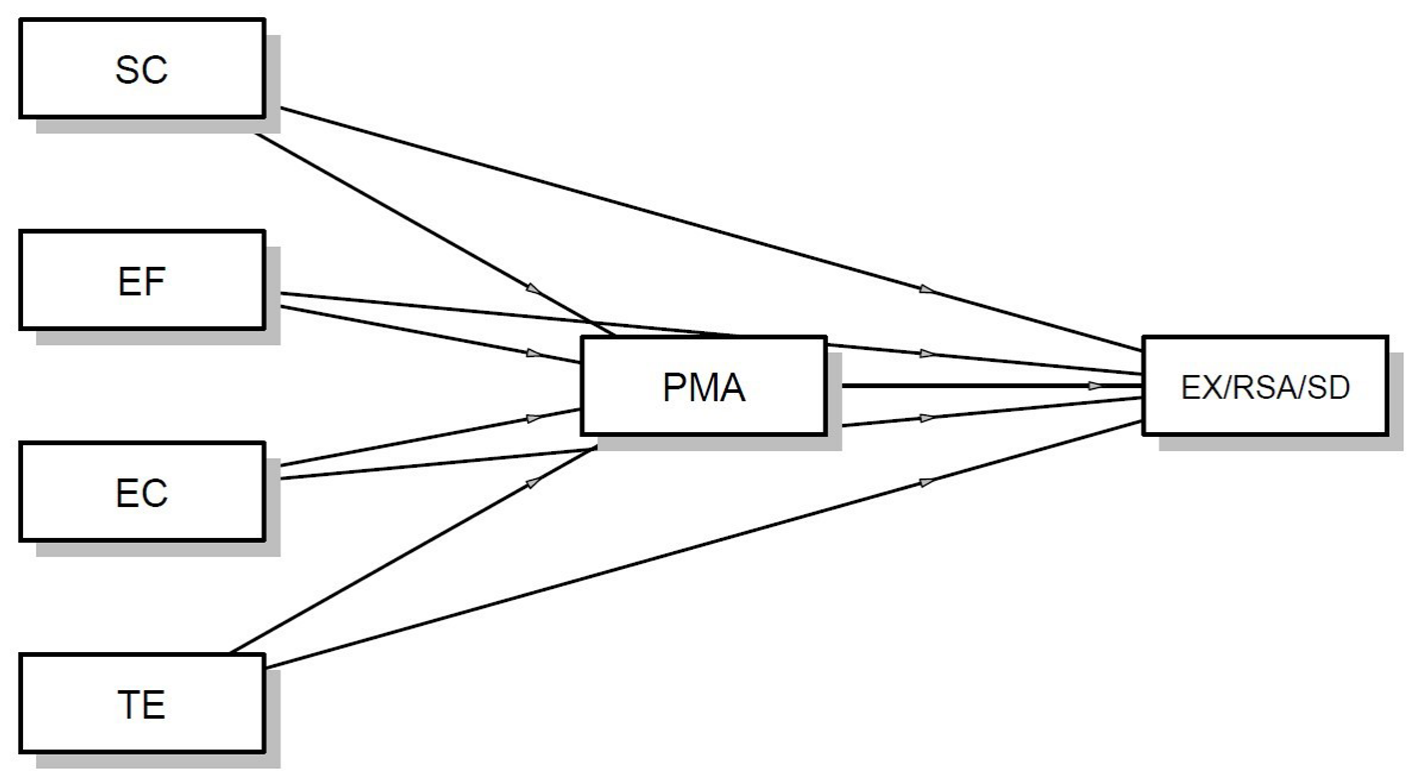
Positive mental attitude as a mediator between components of mental toughness and athlete burnout subscales.

Below is a graphical representation of the mediation model, in which the independent variables are the components of mental toughness, the mediator is positive mental attitude, and the dependent variables are the subscales of athlete burnout (Figure 2).

## 4 Discussion

Athlete burnout is a complex psychophysical phenomenon affecting not only athletes but also other individuals involved in sport-related activities (1, 2). It consists of three components: emotional and physical exhaustion, a reduced sense of accomplishment, and sport devaluation (5). The primary causes of burnout include chronic stress, performance pressure, insufficient recovery, and a lack of environmental support (7, 10). Therefore, preventive efforts should focus on education in stress management, the development of psychological resources, self-regulation skills, and the strengthening of self-efficacy (13, 15). It has also been shown that mental toughness serves as one of the key protective factors against the development of burnout, being associated with higher engagement, better mental health, and more positive interpersonal relationships (29, 35, 36). At the same time, considering that selected components of health-related behaviors (26) may mediate the relationship between mental toughness and burnout (27), this study aimed to determine how the level of mental toughness—through the mediating role of positive mental attitude—relates to the level of burnout among athletes.

The results of this study indicate that a positive mental attitude serves as a significant mediator between mental toughness and athlete burnout. This finding suggests that mental toughness indirectly influences burnout through the development of an important component of health-related behaviors (26). This result can be interpreted within the framework of the Psychological Capital (PsyCap) model derived from positive psychology (37–39), which posits that elements such as hope, optimism, self-efficacy, and mental toughness function as psychological resources that protect the individual. Furthermore, it has been shown that psychological capital influences burnout through coping strategies, which aligns with the observed mediating effect (40).

Studies on athletes have shown that individuals with higher levels of mental toughness cope more effectively with training overload and psychological stress—two of the main predictors of athlete burnout (6, 7). Wu et al. (35) demonstrated that mental toughness moderates the relationship between organizational stressors and burnout among athletes, indicating that individuals with greater mental toughness are less susceptible to burnout despite exposure to stressors. Similarly, findings by Tutte-Vallarino et al. (36) support this relationship, suggesting that developing mental toughness—alongside optimism—is an important preventive strategy against burnout. Kostopoulos et al. (41) further reported that higher mental toughness in basketball players is associated with lower levels of athlete burnout, with positive mental attitude emerging as a key contributing factor.

Furthermore, it has been suggested that mental toughness may facilitate the development of constructive cognitive strategies, which in turn lead to a higher level of positive mental attitude. In our model, positive mental attitude can therefore be seen as an outcome of these adaptive strategies, activated by mental toughness—indicating that mental toughness alone may not be sufficient to reduce burnout levels. In line with this, Turkish studies (42) have shown that components of mental toughness contribute to reducing burnout, with cognitive strategies playing a key mediating role in this relationship.

Our findings further demonstrated that positive mental attitude plays a mediating role in the relationship between selected components of mental toughness—specifically high self-confidence and perceived effectiveness—and two dimensions of athlete burnout: emotional and physical exhaustion and reduced sense of athletic accomplishment. First, these results are consistent with evidence suggesting that individuals with a high level of positive mental attitude are less susceptible to psychological symptoms of burnout (43). Second, it has been proposed that the placement of psychological variables, supported by a positive mental attitude, promotes greater use of task-focused coping strategies. Such strategies may foster sustained engagement and help prevent exhaustion by continuously reinforcing a sense of meaning and purpose in sport (44). Third, the dimension of reduced sense of accomplishment often stems from a subjective perception of insufficient progress, regardless of actual performance. In this context, positive mental attitude may function as a cognitive filter, altering how athletes perceive both success and failure. Individuals with high self-confidence and a strong sense of effectiveness, who also cultivate a positive mental attitude, are more likely to evaluate their performance realistically and constructively (45). Research has also shown that athletes displaying high levels of positive mental attitude tend to rely more on internal standards of success rather than external outcomes, which protects them from undervaluing their achievements (46). Moreover, maintaining a positive mindset helps sustain motivation even during periods of limited progress by reducing self-deprecation (47) and enhancing the perceived value of the developmental process toward athletic goals (44). This, in turn, reinforces a focus on skill mastery rather than purely on results (48).

This study also found that positive mental attitude did not significantly mediate the relationships between the components of mental toughness (self-confidence, effectiveness, emotional control, and task execution) and sport devaluation as a dimension of burnout. In interpreting this result, it may be assumed that sport devaluation, as a component of burnout, may stem from a redefinition of the meaning of sport itself, rather than from a temporary emotional state. While a positive mental attitude may support emotional regulation and motivation maintenance, it may be insufficient to counteract long-term cognitive and existential processes such as the loss of meaning and value associated with sport participation. Setyawati et al. (49) demonstrated that mental toughness does not always translate into a positive narrative about the meaning of sport, particularly when chronic fatigue and disillusionment with the sporting system arise—such as in situations where athletes function within incoherent or toxic environments (50). Furthermore, sport devaluation can be seen as an indicator of declining intrinsic motivation, which is not directly determined by mental attitude but rather by the degree of self-determination in one’s actions. Thus, when an athlete—despite possessing high mental toughness—experiences a deficit in basic psychological needs such as autonomy, competence, and relatedness, positive mental attitude may be insufficient to exert an effective mediating influence (51).

In summary, the findings indicate that mental toughness serves as a protective factor against athlete burnout, and this relationship operates through the mediating role of positive mental attitude. This particular dimension of health-related behavior integrates various psychological resources—such as self-confidence and sense of efficacy—into a cohesive functional system of mental resilience, thereby supporting the athlete’s ability to maintain engagement despite failures and challenges. When an athlete possesses a strong belief in their own abilities under pressure (self-confidence) and trust in the effectiveness of their actions (self-efficacy), these factors can together foster the development of a positive mental attitude (52), which in turn protects against the onset of burnout in sport.

At the same time, we emphasize that the obtained results indicate the need for further, in-depth analyses. First, due to the cross-sectional nature of the present study, future research should employ longitudinal designs to capture the dynamics of athlete burnout over time and to more precisely determine the directionality of relationships between risk factors and the development of burnout symptoms. Second, an important direction for future study is the differentiation of athletic groups and training contexts, which would allow researchers to assess the extent to which the characteristics of training and competitive environments modify the relationships among the examined variables. Another crucial avenue for investigation is the identification of mediators and moderators—such as coping strategies or personal resources—that may explain the variability in burnout levels within athletic populations.

## 5 Conclusion

Based on the conducted research, three main conclusions were formulated:

1. Positive mental attitude serves as a mediator between mental toughness and athlete burnout. Athletes with higher mental toughness tend to display a stronger positive mental attitude, which in turn protects against symptoms of burnout. This finding is consistent with the theoretical framework of psychological capital.
2. Although a positive mental attitude contributes to reducing stress, it appears insufficient to prevent sport devaluation. This limitation likely reflects the fact that devaluation stems from deep-seated beliefs and existential interpretations of sport, which cannot be altered solely through a positive mindset.
3. It is recommended to develop both mental toughness and positive mental attitude—as an integrated structure of psychological resources—as key components of burnout prevention strategies in sport.

## Data Availability

The data analyzed in this study are subject to the following licenses/restrictions: The data are available from the corresponding author on reasonable request. Requests to access these datasets should be directed to Łukasz Bojkowski, bojkowski@awf.poznan.pl.
